# Estimating Risk of Natural Gas Portfolios by Using GARCH-EVT-Copula Model

**DOI:** 10.1155/2015/125958

**Published:** 2015-08-13

**Authors:** Jiechen Tang, Chao Zhou, Xinyu Yuan, Songsak Sriboonchitta

**Affiliations:** ^1^Faculty of Economics, Chiang Mai University, 2397 Suthep, A. Mueang, Chiang Mai 200060, Thailand; ^2^School of Economics, Northwest Normal University, Lanzhou, China; ^3^The People's Bank of China, Zhang Ye City Branch, Zhangye, China; ^4^Faculty of Economics and Management, Yunnan Normal University, Yunnan, China

## Abstract

This paper concentrates on estimating the risk of Title Transfer Facility (TTF) Hub natural gas portfolios by using the GARCH-EVT-copula model. We first use the univariate ARMA-GARCH model to model each natural gas return series. Second, the extreme value distribution (EVT) is fitted to the tails of the residuals to model marginal residual distributions. Third, multivariate Gaussian copula and Student *t*-copula are employed to describe the natural gas portfolio risk dependence structure. Finally, we simulate N portfolios and estimate value at risk (VaR) and conditional value at risk (CVaR). Our empirical results show that, for an equally weighted portfolio of five natural gases, the VaR and CVaR values obtained from the Student *t*-copula are larger than those obtained from the Gaussian copula. Moreover, when minimizing the portfolio risk, the optimal natural gas portfolio weights are found to be similar across the multivariate Gaussian copula and Student *t*-copula and different confidence levels.

## 1. Introduction

Natural gas has become an important commodity for global economy today and is commonly regarded as a strategic commodity. Since the momentous arrival of hedge funds and other new players that have their focus on this strategic commodity, the market for natural gas has become increasingly volatile and risky. Hence, managing the risks associated with spot and futures natural gas prices has become a crucial issue for financial institutions, regulators, and portfolio managers. One of the most popular and usual tools for measuring market risk is VaR. VaR is defined as the amount of loss in a portfolio under given probability over a certain time period [1]. VaR is considered as a benchmark for measuring market risk, which is the main reason that it can summarize risks in a single number. Additionally, CVaR is a supplement to VaR, and it measures the expected loss, given that the loss is greater than or equal to the VaR at certain confidence levels [2]. The purpose of this study is to apply the GARCH-EVT-copula model to estimate the risk measures (VaR and CVaR) for natural gas portfolios.

More and more empirical studies concerned with VaR estimation have been using the copula-GARCH model. Many research literatures have proved that the copula method is a better method to estimate VaR. For example, Huang et al. [3] proposed a new method, conditional copula-GARCH, to measure the VaR of stock portfolios. Their empirical results suggested that conditional copula-GARCH could be quite robust in estimating VaR. Low et al. [4] investigated whether applying copula models to forecast returns for portfolios could produce superior investment performance compared to traditional models. They found that when managing larger portfolios, the Clayton canonical vine copula outperforms the Clayton standard copula. Aloui et al. [5] used the copula-GARCH approach to investigate the conditional dependence structure between crude oil prices and U.S. dollar exchange rates. The empirical results suggested that using the Student *t*-copula improved the accuracy of VaR forecasts. Chen and Tu [6] estimated the risk (VaR) of hedged portfolio to illustrate the potential model risk. They found that copula-based HPVaR outperforms the conventional CCC- and DCC-GARCH estimators. Weiß and Supper [7] estimated the liquidity-adjusted intraday VaR of stock portfolio by using vine copulas for the dependence structure and the ACDP (Autoregressive Conditional Double Poisson) and GARCH processes for the marginal distribution. The empirical evidences showed that the GARCH-Vine copula model performed well in estimating the liquidity-adjusted intraday portfolio profits and losses. Berger [8] used time-varying copulas combined with EVT-based margins. He pointed out that when forecasting the VaR of a portfolio, it is crucial to find the “right” portfolio return distribution. They found that reliable copulas combined with EVT-based margins can capture reliable VaR figures. Aloui et al. [9] investigated the conditional dependence structure between the crude oil and natural gas markets and derived implications for risk management issues related to an oil and gas portfolio within a VaR framework.

The contributions of this paper are twofold. First, to the best of our knowledge, there are no previous empirical papers estimating the risks of natural gas portfolios using the GARCH-EVT-copula model. In this sense, this study can perfectly contribute to this area of the literature. Second, risk measures of natural gas portfolios which are computed by the GARCH-EVT-copula model can be utilized to have implications for natural gas portfolio risk management.

The outline of this paper is as follows. The next section describes the GARCH-EVT-copula model used in the paper.  Section 3 discusses the data and their properties.  Section 4 presents the empirical results of the model and estimate the VaR and the CVaR five-dimensional natural gas portfolios and the optimal natural gas portfolio weights under minimized portfolio risk. The last section delivers the final concluding remarks.

## 2. Data Description

This study focuses on natural gas portfolios consisting of spot and futures prices at the Title Transfer Facility (TTF) Hub. Our dataset consists of daily series of one-day-ahead gas prices (TTFFD), one-month-ahead cash-settled futures natural gas prices (TTFFM), one-quarter-ahead cash-settled futures natural gas prices (TTFFQ), one-season-ahead cash-settled futures natural gas prices (TTFFS), and one-year-ahead cash-settled futures natural gas prices (denoted by TTFFY). The natural gas price series are expressed in euro per megawatt hour (€/MWh). The data are sourced from Datastream and are from January 1, 2007, to May 30, 2014. The data used in this paper are from Monday to Friday, with total of 1934 observations.

**Figure 1:**
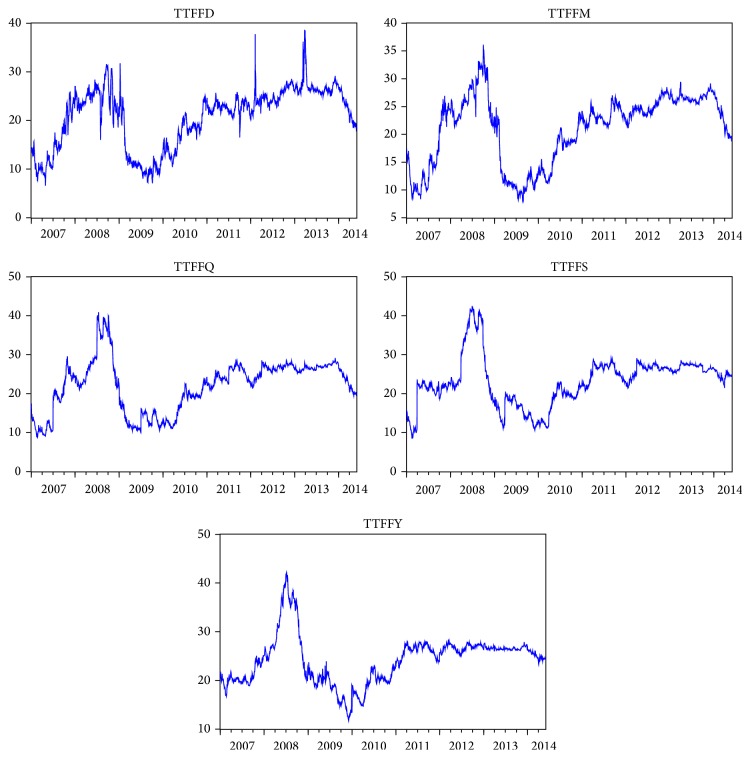
The time series plots of daily natural gas spot and futures prices at the Title Transfer Facility (TTF) Hub.

Figure 1 is plotted to illustrate the natural gas spot and futures prices at the TTF Hub. The figures of the spot and futures prices reveal the typical properties of natural gas prices: extreme price spikes and volatility clustered. The descriptive statistics for natural gas spot and futures returns are reported in Table 1. The natural gas spot and futures returns (*r*_*t*_) are computed on a continuous compounding basis, as *r*_*t*_ = ln⁡(*P*_*t*_/*P*_*t*−1_), where *P*_*t*_ and *P*_*t*−1_ are current and one-period lagged daily natural gas spot or futures prices. The mean returns for all spot and futures series are very small and near zero, indicating that there is no significant trend in the data. All the returns exhibit significant skewness, except for TTFFD and kurtosis. All the returns exhibit positive skewness except for TTFFD. Second, all the returns strongly reject the null hypotheses for the JB (Jarque-Bera) test of normality. Finally, augmented Dickey-Fuller [10] and Kwiatkowski-Phillips-Schmidt-Shin [11] tests are used for the unit root test. Table 1 shows the results, indicating that all return series are stationary at the 1% significance level.

## 3. Methodology

### 3.1. Model for Marginal Distribution

We adopt the univariate standard GARCH model for natural gas price to obtain our marginal distributions in this study. The univariate standard GARCH(1, 1) model [12] is defined as follows:

(1) $$\begin{array}{c} r_{s,t}=c_{s,0}+\overset{p}{\underset{i=1}{\sum }}\phi_{s,i}r_{s,t-i}+\overset{q}{\underset{j=1}{\sum }}\theta_{s,j}r_{s,t-j}+e_{s,t},\\ e_{s,t}=\sqrt{h_{s,t}}z_{s,t},\\ h_{s,t}=\omega_{s}+\alpha_{s}e_{s,t-1}^{2}+{\beta_{s}h}_{s,t-1}, \end{array}$$

where *s* is TTFFD, TTFFM, TTFFQ, TTFFS, and TTFFY, respectively, *r*_*s*,*t*_ denotes the spot or futures return of natural gas, *h*_*s*,*t*_ is the conditional variance of volatility, *ϕ*_*s*,*i*_ is the *i*th lag AR parameter, *θ*_*s*,*j*_ is the *i*th lag MA parameter, *α*_*s*_ is the ARCH parameter, and *β*_*s*_ is the GARCH parameter. The conditions *ω*_*s*_ > 0, *α*_*s*_, *β*_*s*_ ≥ 0, and *α*_*s*_ + *β*_*s*_ < 1 are to ensure that the conditional variance process remains positive and stationary. *z*_*s*,*t*_ is the standardized error. In Section 2, we know that all series are not normal distribution. Hence, we assume that *z*_*s*,*t*_ is *t*-distribution. df_*s*_ is the degree of freedom, which is denoted as *λ*_*s*_. In order to better estimate the tails of the distribution, this paper applied EVT to those residuals. We model residuals by using the generalized Pareto distribution (GPD) estimate for the upper and lower tails and the Gaussian kernel estimate for the remaining part. The CDF of a GPD distribution is parameterized as follows:

(2) Fzs,t =NmLN1+ξL(mL−zs,t)βL−1/ξL,zs,t<mL,φzs,t,mL<zs,t<mR,1−NmRN1+ξR(mR−zs,t)βR−1/ξR,zs,t>mR,

where *ξ* is the shape parameter, *β* is the scale parameter, and *m*_*L*_(*m*_*R*_) is the lower (upper) threshold.

There is a trade-off between high precision and low variance; the critical step is the choice of the optimal threshold. Following DuMouchel [13], this paper chose the exceedances to be the 10th percentile of the sample.

### 3.2. Copula

Let *X*_1_,…, *X*_*n*_ be the random variables with the marginal distribution *F*_1_,…, *F*_*n*_. Then, there exists a copula *C* : [0,1]^*n*^ → [0,1] that satisfies the following function:

(3) $$\begin{array}{c} F\left(x_{1},\ldots ,x_{n}\right)=C\left(F_{1}\left(x_{1}\right),{\ldots ,F}_{n}\left(x_{n}\right)\right)=C\left(u_{1},\ldots ,u_{n}\right), \end{array}$$

where *u*_*i*_ = *F*_*i*_(*x*_*i*_). If the marginal distribution *F*_*i*_(*x*_*i*_) is continuous, then *C* is unique; otherwise, *C* is uniquely determined by Ran*F*_1_ × Ran*F*_2_ × ⋯×Ran*F*_*n*_.

The multivariate Gaussian copula [14] is given as

(4) C∑ρGauu1,t,u2,t,…,un,t ∣ ρ =Φ∑ρΦ−1u1,t,Φ−1u2,t,…,Φ−1un,t,

where *u*_*n*,*t*_ is the cumulative distribution function of the standardized error from the marginal models, Φ_∑*ρ*_ denotes the standardized multivariate normal distribution with the correlation matrix ∑*ρ*, Φ^−1^ is the inverse of the univariate standard normal distribution, and *ρ* ∈ (−1,1) measures the dependence.

The Gaussian copula cannot capture the dependence of a fat tail. In order to capture the fat tail, we also introduced the multivariate Student *t*-copula in this study. The multivariate Student *t*-copula [14] is defined as

(5) C∑ρ,vStuu1,t,u2,t,…,un,t ∣ ρ,n   =t∑ρ,vtv−1u1,t,tv−1u2,t,…,tv−1un,t,

where *t*_∑*ρ*,*v*_ is the standardized multivariate Student *t*-distribution with correlation and degrees of freedom matrix ∑*ρ*, *v*; correlation *ρ* ∈ (−1,1),   *t*_*v*_^−1^ denotes the inverse of the univariate Student *t*-distribution. The parameters of *v* and *ρ* determine the extent of symmetric extreme dependence by the upper and lower tail dependences, *λ*_*L*_ and *λ*_*R*_, such that $\lambda_{L}=\lambda_{R}=2t_{v+1}\left(-\left(\left(\sqrt{v+1}\sqrt{1-\rho }\right)/\sqrt{1+\rho }\right)\right)>0$.

### 3.3. Portfolio Risk Analysis

#### 3.3.1. VaR and CVaR

In order to analyze risk management, this paper used VaR and CVaR to measure the risk. We estimate the VaR and the CVaR by taking the *α*-quantiles of the portfolio return forecasts, as given in the following equation:

(6) $$\begin{array}{c} {VaR}_{t+1\;|\;t}\left(\alpha \right)=F^{-1}\left(q\right){\hat{r}}_{t+1,P},\\ C{VaR}_{t+\left(1/t\right)}\left(\alpha \right)=E\left({\hat{r}}_{t+1,p}\;|\;{\hat{r}}_{t+1,p}>{VaR}_{t+1\;|\;t}\left(\alpha \right)\right). \end{array}$$

We assume that the weight in each asset is the same; that is, *ω* = [(1/5), (1/5), (1/5), (1/5), (1/5)]′. As a result, we can compute the VaR and the CVaR of the equally weighted portfolio.

#### 3.3.2. Optimal Portfolio with Minimum Risk

We estimate the VaR and the CVaR of an equally weighted portfolio, but the major concern for commercial banks and individual investors is to minimize the risk of the investment portfolio. To address this concern, in this paper, we also compute the optimal weights of each asset that minimize the portfolio risk. According to Wang et al. [2], the algorithm is as follows. We assume that the weight in individual asset within a portfolio is *ω* = [*ω*_1_, *ω*_2_, *ω*_3_, *ω*_4_,*ω*_5_]′, where 0 ≤ *ω*_*s*_ ≤ 1  (*s* = 1, 2,…, 5) and *ω*_1_ + *ω*_2_ + *ω*_3_ + *ω*_4_ + *ω*_5_ = 1. Many weight vectors can satisfy these conditions. Given a weight vector  *ω*, *N* returns of the portfolio are obtained; this means that returns = *r*_*t*+1_ × *ω*. Subsequently, the VaR of these portfolios at a given confidence level can be computed. After getting all the VaR values corresponding to all the weight vectors, the minimum VaR can be calculated. Thus, we can get the weight of each asset that minimizes the VaR; that is, min⁡VaR_*α*_ = risk(*b*_1_, *b*_2_, *b*_3_, *b*_4_, *b*_5_).

### 3.4. Estimation Procedure

We follow a seven-step estimation procedure in this paper.

(1) We first complete the parameter estimation and obtain the standardized residuals by fitting the univariate ARMA(*p*, *q*)-GARCH(1,1) model for each natural gas return series.
(2) For EVT, we chose the exceedances to be the 10th percentile of the sample and used the sample MEF plot and the Hill plot to determine an appropriate threshold for the upper and lower tails of the distribution of the residuals. We assume that excess residuals over this threshold follow the generalized Pareto distribution (GPD) and estimate for the upper and lower tails and use the Gaussian kernel estimation for the remaining part.
(3) We transform the standardized residuals *z*_*s*,*t*_ from the GARCH model into the variates *u*_*s*,*t*_, using the ECDFs (empirical cumulative distribution functions). Thereafter, we perform the goodness-of-fit test for each *u*_*s*,*t*_.
(4) We fit a copula model and estimate its parameter.
(5) We simulate *N* times from the estimated copula model to convert to *N* standardized residuals using the inverse of the corresponding distribution function for each model.
(6) We forecast *N* portfolio returns using the ARMA(*p*, *q*)-GARCH(1,1) model.
(7) We estimate the VaR and the CVaR of the equally weighted portfolio. Additionally, we compute the optimal portfolio with the minimum risk.

## 4. Empirical Results

### 4.1. Results for Marginal Model

Firstly, estimate the ARMA(*p*, *q*)-GARCH(1,1) model for each natural gas return. The AIC, BIC, and Loglik statistics are adopted to select the most suitable models. The estimated coefficients and standard errors for the marginal model are presented in Table 2. Table 2 shows that the AR(1) estimates *ϕ*_*s*,1_ are significant in all the series, except for TTFFM and TTFFQ, which implies that the current returns have spillover effects from their returns in one of the previous periods. Second, it can be observed that the ARCH parameter *α*_*s*_ and the GARCH parameter *β*_*s*_ are statistically significant, implying that all the returns experience ARCH and GARCH effects. The *P* values of ARCH(10) are more than 10%, which shows that there is no heteroskedasticity effect in the residuals. Additionally, the degrees of freedom *λ*_*s*_ of the Student *t*-distribution for all the returns are significant, suggesting that the error terms are not normal distribution and that the Student *t*-distribution works reasonably well for all the return series.

As described in Section 3.1, we apply EVT to those residuals. GPD specially is for tail estimation, and we choose the exceedances to be the 10th percentile of the sample. The Gaussian kernel estimate is for the remaining part. The parameter estimation for each natural gas's residuals is demonstrated in Table 3.

Given the availability of the estimates of marginal models, we proceed to estimate the copula functions. For that, we transform the standardized residuals *z*_*s*,*t*_ from the GARCH model into the variates *u*_*s*,*t*_, using the ECDF. Each variate *u*_*s*,*t*_ should be uniform (0, 1); otherwise, the copula model could be misspecified. This paper follows Patton [15] and Reboredo [16] to carry out the goodness-of-fit test, which are Ljung-Box (LB) test and Kolmogorov-Smirnov (KS) test. LB test is used to examine the serial correlation under the null hypothesis of serial independence. To do so, ${\left(u_{s,t}-{\overset{-}{u}}_{s}\right)}^{k}$ is regressed on the first 10 lags of the variables. And KS test is used to test the null hypothesis that the *u*_*i*,*t*_ are uniform (0, 1).  Table 4 reports the *P* values of these two tests. At the 5% significance level, all the null hypotheses are rejected, which implies that the marginal distribution model demonstrates that these are not misspecified. Hence, it can be concluded that the copula model can correctly capture the dependence.

### 4.2. Results for Copula

After estimating the parameters of the marginal distribution, we proceed further to estimate the copula parameter. The multivariate Gaussian copula and Student *t*-copula are applied in this study. The dependence matrix is estimated by the maximum-likelihood estimation (MLE) method. The results of the dependence matrix for the multivariate Gaussian copula model and the Student *t*-copula model are reported in Tables 5 and 6, respectively. The dependence parameters are positive and strongly significant at 10% level for the Gaussian and the Student *t*-copula models. These findings point out the presence of significantly strong positive relationships between the TTF natural gas portfolios. Second, the degree of freedom for the multivariate Student *t*-copula *v* is reasonably low (4.9837), which suggests the existence of substantially extreme comovements and tail dependence for all the pairs of the TTF natural gas portfolios. This finding indicates that extreme events or crises are likely to spread from one series to another during the bull and bear periods.

### 4.3. Portfolio Risk Analysis

After modeling the dependence structure, we simulate one-month natural gas portfolio returns based on the dependence structure specified in both the multivariate Gaussian copula and the Student *t*-copula and estimate the risk of a five-dimensional natural gas portfolio. With an equally weighted portfolio of five natural gas returns (TTFFD, TTFFM, TTFFQ, TTFFS, and TTFFY), we can calculate the VaR and the CVaR of the portfolio. The results of the VaR and the CVaR calculations are presented in Table 7. From Table 7, we can perceive that the VaR and the CVaR calculated from the Student *t*-copula are more than those from the Gaussian copula under the same confidence level. The reason is that the Student *t*-copula considers the extreme dependence, whereas the Gaussian copula does not.

Likewise, in this paper, we also compute the optimal weights of each asset that minimize the portfolio risk. The optimal portfolio weight under the minimum portfolio risk and the VaR (CVaR) under different copulas and confidence levels are presented in Table 8. From Table 8, we can draw the following interesting conclusions.

(1) The optimal portfolio weights are similar across the multivariate Gaussian copula and Student *t*-copula and different confidence levels. The optimal investment tends to concentrate on TTFFM, TTFFQ, TTFFS, and TTFFY. Moreover, the optimal portfolio weights of TTFFM, TTFFQ, TTFFS, and TTFFY are almost equal. However, the optimal portfolio weight of TTFFD is nearly zero. This is because TTFFD is the spot (one-day-ahead) price, and TTFFM, TTFFQ, TTFFS, and TTFFY are the futures prices. Futures markets have risk aversion function. Under minimum portfolio risk, investors prefer the futures market. On the other hand, with higher confidence levels, the weight of TTFFD investment becomes larger. The reason is that, with higher confidence levels, investors will be more risk tolerant and, therefore, willing to take more risk to achieve higher expected returns.
(2) Comparing the risk value calculated by the Gaussian copula and Student *t*-copula, we observe that most of the VaR and all the CVaR from the Gaussian copula are less than those from the Student *t*-copula. This result is consistent with the conclusions when computing the VaR and the CVaR of the equally weighted foreign exchange portfolio. This is because the Student *t*-copula, on account of its heavy tail, is able to capture the extreme dependence. The extreme events of the asset returns have higher dependence and hence larger VaR and CVaR.

## 5. Conclusion

We employed the GARCH-EVT-copula model to estimate the risk of a multidimensional natural gas portfolio. This study focuses on the natural gas portfolio at the Title Transfer Facility (TTF) Hub. The data consist of daily series of one-day-ahead gas prices (TTFFD), one-month-ahead cash-settled futures natural gas prices (TTFFM), one-quarter-ahead cash-settled futures natural gas prices (TTFFQ), one-season-ahead cash-settled futures natural gas prices (TTFFS), and one-year-ahead cash-settled futures natural gas prices (denoted by TTFFY). Our dataset is from January 1, 2007 to May 30, 2014. The multivariate Gaussian copula and Student *t*-copula have been employed to model the dependence structure.

The empirical results show that, for an equally weighted portfolio of five natural gases, the VaR and the CVaR obtained from the Student *t*-copula are larger than those obtained from the Gaussian copula. The reason is that the Gaussian copula cannot capture the extreme dependence but the Student *t*-copula can. Second, the optimal portfolio weights are found to be similar across the multivariate Gaussian copula and Student *t*-copula and different confidence levels upon minimizing the portfolio risk. The optimal investment tends to concentrate on TTFFM, TTFFQ, TTFFS, and TTFFY. In addition, the optimal portfolio weights of TTFFM, TTFFQ, TTFFS, and TTFFY are almost equal. At the same time, the optimal portfolio weight of TTFFD is nearly zero. With higher confidence levels, the weight of the TTFFD investment becomes larger.

Further studies on estimating the risk of natural gas portfolios should concentrate on modeling dependence. Specifically, we should employ more copulas: multivariate Clayton, multivariate Gumbel, and vine copula. Furthermore, the risk of natural gas portfolio in different Hubs should be considered. Third, we should work on backtesting to check the performance of the approach compared to other popular approaches of VaR estimation. We intend to address these matters in our future research.

## Figures and Tables

**Table 1:** Descriptive statistics of spot and generic futures returns for natural gas portfolio.

	TTFFD	TTFFM	TTFFQ	TTFFS	TTFFY
Mean	0.0002	0.0000	0.0001	0.0002	0.0001
Median	0.0000	0.0000	−0.0007	−0.0005	0.0000
Maximum	0.3923	0.2672	0.5462	0.7724	0.3476
Minimum	−0.3538	−0.1171	−0.2027	−0.1780	−0.3482
Std. dev.	0.0453	0.0273	0.0286	0.0289	0.0214
Skewness	−0.0396	1.7707^**^	6.3836^**^	12.5263^**^	2.3924^**^
Kurtosis	11.7180^**^	18.1748^**^	111.4036^**^	306.0295^**^	115.0176^**^
JB	6125.1020^**^	19566.9600^**^	960097.8000^**^	7450294.0000^**^	1013000.0000^**^
ADF	−49.3317^**^	−27.2174^**^	−43.5283^**^	−42.2873^**^	−27.6217^**^
KPSS	0.05345	0.0805	0.07311	0.04966	0.0614
Observations	1934	1934	1934	1934	1934

*Note.* ∗∗ denotes rejection of the null hypothesis at the 1% and 5% significance levels, respectively.

**Table 2:** Estimated parameters of agricultural commodity returns for marginal model.

	TTFFD	TTFFM	TTFFQ	TTFFS	TTFFY
*c*_*s*,*o*_	−0.0002 (0.0004)	−0.0007^**^ (0.0003)	−0.0008 (0.0003)	−0.0005^*^ (0.0002)	−0.0003 (0.0002)

*ϕ*_s,1_	−0.1350^**^ (0.0234)	0.0363 (0.0206)	0.0206 (0.0190)	0.0668^**^ (0.0218)	0.0588^**^ (0.0223)

*ω*_*s*_	0.0000^**^ (0.0000)	0.0000 (0.0000)	0.0000^**^ (0.0000)	0.0000^**^ (0.0000)	0.0000^**^ (0.0000)

*α*_*s*_	0.2045^**^ (0.0263)	0.0560^**^ (0.0169)	0.0120^**^ (0.0035)	0.1865^**^ (0.0341)	0.1661^**^ (0.0243)

*β*_*s*_	0.7945^**^ (0.0238)	0.9430^**^ (0.0170)	0.9848^**^ (0.0030)	0.8125^**^ (0.0305)	0.8329^**^ (0.0213)

*λ*_*s*_	4.3963^**^ (0.3857)	3.2386^**^ (0.2172)	2.8659^**^ (0.1965)	3.1684^**^ (0.1866)	3.6138^**^ (0.2616)

LL	3910.0000	4943.7790	5154.1850	5409.1680	5794.5190

AIC	−4.0372	−5.1063	−5.3239	−5.5876	−5.9861

ARCH(10)	0.7074	0.8258	0.9988	1.0000	0.9996

*Note.* The table shows the estimates and the standard errors of the parameters for the marginal distribution model defined in (3) and (4). ∗∗ and ∗ denote rejection of the null hypothesis at the 1% and 5% significance levels, respectively. ARCH(10) is the *P* value of Engle's LM test for the ARCH effect in the residuals up to the 10th order.

**Table 3:** Parameter estimation for each natural gas's residuals.

	TTFFD	TTFFM	TTFFQ	TTFFS	TTFFY
Threshold *m*_*L*_	−1.2278	−1.0081	−1.0463	−1.0491	−1.0857
*ξ*_*L*_	0.0489	0.0588	0.1494	0.2596	0.0817
*β*_*R*_	0.7085	0.5076	0.4999	0.5140	0.5243
Threshold *m*_*R*_	1.1688	1.0944	1.1152	1.0989	1.1696
*ξ*_*R*_	0.1999	0.3183	0.5386	0.4963	0.2987
*β*_*R*_	0.5327	0.6823	0.5883	0.5606	0.6718

**Table 4:** Goodness-of-fit test for marginal distributions.

	TTFFD	TTFFM	TTFFQ	TTFFS	TTFFY
First moment LB test	0.4000	0.1278	0.2771	0.2530	0.1008
First moment LB test	0.2171	0.6031	0.0562	0.0862	0.1753
First moment LB test	0.5548	0.0791	0.4020	0.0503	0.1164
First moment LB test	0.1283	0.0651	0.1357	0.1247	0.2840
KS test	0.9997	1.0000	0.9436	0.9997	0.8967

*Note.* This table reports the *P* values from the Ljung-Box (LB) test for serial independence of the first four moments of the variable u_s,t_. We regress ${\left(u_{s,t}-{\overset{-}{u}}_{s}\right)}^{k}$ on the first five lags of the variables for *k* = 1,2, 3,4. In addition, we present the *P* values of the Kolmogorov-Smirnov (KS) test for the adequacy of the distribution model.

**Table 5:** Estimated parameters of natural gas portfolio for multivariate Gaussian copula.

	TTFFD	TTFFM	TTFFQ	TTFFS	TTFFY
TTFFD	1.0000	0.5247^**^ (0.0146)	0.4945^**^ (0.0156)	0.4203^**^ (0.0172)	0.3772^**^ (0.0178)

TTFFM	0.5247^**^ (0.0146)	1.0000	0.6884^**^ (0.0098)	0.6780^**^ (0.0102)	0.6258^**^ (0.0114)

TTFFQ	0.4945^**^ (0.0156)	0.6884^**^ (0.0098)	1.0000	0.7018^**^ (0.0056)	0.6864^**^ (0.0099)

TTFFS	0.4203^**^ (0.0172)	0.6780^**^ (0.0102)	0.7018^**^ (0.0056)	1.0000	0.8250^**^ (0.0095)

TTFFY	0.3772^**^ (0.0178)	0.6258^**^ (0.0114)	0.6864^**^ (0.0099)	0.8250^**^ (0.0095)	1.0000 (0.0095)

*Note.* ∗∗ denotes rejection of the null hypothesis at the 1% and 5% significance levels, respectively.

**Table 6:** Estimated parameters of natural gas portfolio for the multivariate Student *t*-copula.

*v* = 4.9837	TTFFD	TTFFM	TTFFQ	TTFFS	TTFFY
TTFFD	1.0000	0.5525^**^ (0.0161)	0.4995^**^ (0.0177)	0.4408^**^ (0.0190)	0.4003^**^ (0.0194)

TTFFM	0.5525^**^ (0.0161)	1.0000	0.7755^**^ (0.0087)	0.7681^**^ (0.0089)	0.7071^**^ (0.0107)

TTFFQ	0.4995^**^ (0.0177)	0.7755^**^ (0.0087)	1.0000	0.7830^**^ (0.0084)	0.8753^**^ (0.0089)

TTFFS	0.4408^**^ (0.0190)	0.7681^**^ (0.0089)	0.7830^**^ (0.0084)	1.0000	0.7514^**^ (0.0093)

TTFFY	0.4003^**^ (0.0194)	0.7071^**^ (0.0107)	0.8753^**^ (0.0089)	0.7514^**^ (0.0093)	1.0000

*Note.* ∗∗ denotes rejection of the null hypothesis at the 1% and 5% significance levels, respectively.

**Table 7:** Portfolio risk under equally weighted natural gas portfolio.

Confidence level	Risk value	Normal copula	*t*-copula
0.90	VaR	0.0746	**0.0755**
	CVaR	0.1094	**0.1112**

0.95	VaR	0.0977	**0.0991**
	CVaR	0.1344	**0.1365**

0.99	VaR	0.1565	**0.1624**
	CVaR	0.1989	**0.1994**

**Table 8:** Optimal investment proportion of natural gas portfolio with minimum risk.

Copula	Confidence level	VaR	Corresponding CVaR	Optimal coefficients				
				TTFFD	TTFFM	TTFFQ	TTFFS	TTFFY
Normal copula	0.90	0.0130	0.0154	0.0001	0.2499	0.2500	0.2500	0.2500
	0.95	0.0169	0.0189	0.0001	0.2498	0.2500	0.2500	0.2500
	0.99	0.0260	0.0274	0.0158	0.2389	0.2453	0.2450	0.2500

Student *t*-copula	0.90	0.0126	0.0159	0.0001	0.2498	0.2499	0.2501	0.2500
	0.95	0.0171	0.0195	0.0001	0.2499	0.2499	0.2501	0.2500
	0.99	0.0280	0.0287	0.0064	0.2468	0.2480	0.2492	0.2496
